# Looking for the ideal medication for heart failure with reduced ejection fraction: a narrative review

**DOI:** 10.3389/fcvm.2024.1439696

**Published:** 2024-09-06

**Authors:** Domingo Pascual-Figal, Antoni Bayes-Genis

**Affiliations:** ^1^ Cardiology Department, Hospital Universitario Virgen de la Arrixaca, Murcia, Spain; ^2^ University of Murcia, Murcia, Spain; ^3^ Centro Nacional de Investigaciones Cardiovasculares (CNIC), Madrid, Spain; ^4^ Cardiology Service, Hospital Universitari Germans Trias i Pujol, Badalona, Spain

**Keywords:** heart failure with reduced ejection fraction, angiotensin-converting enzyme inhibitors, angiotensin II receptor blockers, beta-blockers, mineralocorticoid receptor antagonists, sodium-glucose co-transporter 2 inhibitors, angiotensin receptor-neprilysin inhibitors

## Abstract

The main goals of the pharmacological treatment of Heart failure with reduced ejection fraction (HFrEF) are the reduction of mortality and the prevention of hospitalizations. However, other outcomes such as improvements in cardiac remodeling and clinical status, functional capacity and quality of life, should be taken into account. Also, given the significant inter-individual and intra-individual variability of HF, and the fact that patients usually present with comorbidities, an appropriate treatment for HFrEF should exert a clinical benefit in most patient profiles irrespective of their characteristics or the presence of comorbidities, while providing organ protection beyond the cardiovascular system. The aim of this narrative review is to determine which are the proven effects of the guideline-directed treatments for HFrEF on five key clinical outcomes: cardiovascular mortality and hospitalization due to HF, sudden death, reverse cardiac remodeling, renal protection and evidence in hospitalized patients. Publications that fulfilled the pre-established selection criteria were selected and reviewed. Renin-angiotensin system (RAS) inhibitors, namely angiotensin-converting enzyme inhibitors (ACE-I) and angiotensin II receptor blockers (ARBs) or angiotensin receptor-neprilysin inhibitors (ARNI), beta-blockers (BB), mineralocorticoid receptor antagonists (MRA), sodium-glucose co-transporter 2 inhibitors (SGLT2i) show a benefit in terms of mortality and hospitalization rates. ARNI, BB, and MRA have demonstrated a significant positive effect on the incidence of sudden death. ARB, ARNI, BB and SGLT2i have been associated with clear benefits in reverse cardiac remodeling. Additionally, there is consistent evidence of renal protection from ARB, ARNI, and SGLT2i in renal protection and of benefits for hospitalized patients from ARNI and SGLT2i. In conclusion, the combination of drugs that gather most beneficial effects in HFrEF, beyond cardiovascular mortality and hospitalization, would be ideally pursued.

## Introduction

Hearth failure (HF) with reduced ejection fraction (HFrEF) is characterized by a left ventricular ejection fraction (LVEF) of ≤40% (1). HFrEF accounts for 66% of new-onset HF cases (2), and incidence rates are lower in women than in men (3). In a study of more than 2,300 ambulatory patients with HFrEF with an all-cause mortality rate of 33%, around 75% of patients died due to HF-related factors. This rate was lower in patients with LVEF over 40% (4). HF also contributes to the hospitalization burden: within 5 years after the diagnosis, 83% of patients with HF are hospitalized at least once, and 43% are hospitalized four or more times (5). Hospitalization, in turn, reduces survival rates vs. general population, and this effect is even greater in recurrently hospitalized patients (6). Additionally, hospitalization accounts for 76% of HF-associated costs (7). Also, the risk of sudden death (SD) among patients with HFrEF, although decreasing over time with the increasing use of evidence-based medications (8), still remains a major factor. In light of these data, two of the goals of the pharmacological treatment are the reduction of mortality and the prevention of recurrent hospitalizations (1, 9).

However, other outcomes should be taken into account when choosing the most appropriate drug in patients with HFrEF. Current guidelines state that improvement in clinical status, functional capacity, and quality of life of patients are the third goal of pharmacotherapy (1). HF is a dynamic condition and patients exhibit inter-individual and intra-individual variability, and differences are observed throughout the course of the disease (10). Consequently, the most appropriate treatment should exert a clinical benefit in most patient profiles and be efficient irrespective of patient characteristics (sex, age, etiology, LVEF, or the presence of comorbidities, among others). Patients with HF have a high comorbidity burden (11, 12), so the most appropriate treatment should provide organ protection beyond the cardiovascular system. Finally, adherence and appropriate dosing are crucial for achieving clinical benefit and protection and are directly related to survival and the reduction of HF-associated hospitalizations (13, 14).

In this article we review the suitability of each pharmacotherapeutic option for the treatment of patients with HFrEF, considering the four pillars based on guideline-directed medications: RAS blockers (ACE-I, ARB) or ARNI, BB, MR and SGLT2i.

## Methods

For this narrative review we searched in the PubMed database merging our therapeutic classes of interest and the described clinical outcomes. The terms used for the search were ACEi, ARB, ARNI, BB, MRA and SGLT2i, in combination with cardiovascular mortality and HF, hospitalization and HF, SD and HF, reverse cardiac remodelling and HF, renal protection and HF or hospitalized patients and HF. We have analyzed a total of 48 studies of which 32/48 (66.6%) were double-blind randomized clinical trials (24 placebo-controlled, 8 of them controlled with an active comparator), 2/48 (4.2%) was an open-label randomized clinical trial, 9/48 (18.8%) were *post hoc* analysis of randomized clinical trials, 3/48 (6.3%) were cohort studies and 2/48 (4.2%) were meta-analysis.

## Results

### Effects of pharmacological treatment for heart failure on cardiovascular mortality and hospitalization due to heart failure

All approved therapeutic options for HFrEF exert a positive effect on cardiovascular mortality and hospitalization due to HF (Table 1). The CONSENSUS trial evaluated the effect of the ACEi enalapril compared to placebo on the prognosis of severe congestive HF. Conventional treatment for HF continued in both groups. After 6 months of treatment, a 40% reduction of total mortality was observed in the enalapril arm (*P* = 0.002). Mortality due to progression of HF also decreased by 50% in the enalapril arm (*P* < 0.001) (15). Regarding the effect of enalapril on hospitalization, the SOLVD trial showed that the addition of enalapril to conventional therapy produced a 26% reduction in the rate of events after 48 months, defined as either death or hospitalization of patients, compared to placebo (*P* < 0.0001) (16).

**Table 1:** Effect of pharmacological treatment on cardiovascular mortality and hospitalization due to heart failure.

Publication	Intervention	Control	Endpoints	Results	N/demographic characteristics	Summary of the effect
ACEi						
CONSENSUS trial study Group. N Engl J Med. 1987 (15)	Enalapril	Placebo	Mortality from any cause at 6 months	Risk reduction 40% (*P* = 0.002)	*N* = 253 patients, NYHA class IV cHF	Evidence showing benefit
			Mortality due to HF worsening	Risk reduction 50% (*P* < 0.001)		
SOLVD investigators. N Engl J Med. 1991 (16)	Enalapril	Placebo	Death or hospitalization after 48 months	Risk reduction 26% (*P* < 0.0001)	*N* = 2,569 patients <80 years, NYHA class II or III cHF, EF ≤0.35	
			CV deaths	Risk reduction 18% (*P* < 0.002)		
ARB						
Val-HeFT trial Cohn JN, et al. N Engl J Med. 2001 (17)	Valsartan	Placebo	Combined mortality and morbidity	Risk reduction 13% (*P* = 0.009), driven by 13.8% decrease in hospitalization (*P* < 0.001)	*N* = 5,010 patients, NYHA class II, III or IV HF	Evidence showing benefit
CHARM-Added trial. McMurray JJ et al. Lancet. 2003. (18)	Candesartan	Placebo	Composite of cardiovascular death or hospital admission for congestive HF	Unadjusted HR 0.85, 95% CI 0.75–0.96, *P* = 0.011; covariate adjusted *P* = 0.010	*N* = 2,548 patients LVEF≤ 40% and already on ACEi	
CHARM-Alternative trial Granger CB et al. Lancet. 2003. (19)	Candesartan	Placebo	Composite of cardiovascular death or hospital admission for congestive HF	Unadjusted HR 0.77, 95% CI 0.67–0.89, *P* = 0.0004; covariate adjusted 0.70 [0.60–0.81], *P* < 0.0001)	*N* = 2,028 patients LVEF ≤ 40% and ACE intolerant	
ARNI						
McMurray J, et al. N Engl J Med. 2014 (20)	Sacubitril/valsartan	Enalapril	Death from CV causes or first hospitalization for worsening HF	HR 0.80 (95% CI 0.73, 0.87; *P* < 0.001)	*N* = 8,442 patients, class II, III or IV HF, EF≤40%	Evidence showing benefit
			Death from cardiovascular causes	HR 0.80 (95% CI 0.71, 0.89; *P* < 0.001)		
			First hospitalization for worsening HF	HR 0.79 (95% CI 0.71, 0.89; *P* < 0.001)		
Packer M, et al. Circulation. 2015 (21)	Sacubitril/valsartan	Enalapril	Hospitalizations for worsening HF	HR 0.79 (95% CI 0.71, 0.89; *P* < 0.001)	*N* = 8,339 patients, NYHA class II-IV HF, EF ≤ 40%	
Velazquez EJ, et al. N Engl J Med. 2019 (22)	Sacubitril/valsartan	Enalapril	Change in NT-proBNP	HR 0.71 (95% CI 0.63, 0.81; *P* < 0.001)	*N* = 881 patients ≥ 18 years, LVEF ≤ 40%, acute decompensated HF	
*Post hoc* analysisi of TRANSITION Pascual-Figal D, et al. JACC Heart Fail. 2020 (23)	Sacubitril/valsartan pre-discharge	Sacubitril/valsartan post-discharge	Change in NT-proBNP	−28.1% versus −3.5% (*P* < 0.001)	*N* = 991 patients ≥ 18 years, mean 66.8 years, 75.1% male, hospitalized for an ADHF, LVEF ≤ 40%, NYHA class II-IV	
Beta-blockers						
Packer M, et al. N Engl J Med. 1996 (24)	Carvedilol	Placebo	Mortality after 12 months	Risk reduction 65% (95% CI, 39–80%; *P* < 0.001	*N* = 1,904 patients, chronic HF, LVEF ≤ 0.35	Evidence showing benefit
			Hospitalization for cardiovascular causes	Risk reduction 27% (95% CI 39%, 80%; *P* < 0.001)		
			Death or hospitalization	Risk reduction 38% (95% CI 18%, 53%; *P* < 0.001)		
CIBIS-II Investigators and Committees. Lancet. 1999 (25)	Bisoprolol	Placebo	CV mortality	HR 0.71 (95% CI 0.56, 0.90; *P* = 0.0049)	*N* = 2,647 patients, NYHA class III or IV, LVEF≤35%, receiving diuretics and ACEi	
			Hospital admission for worsening HF	HR 0.64 (95% CI 0.53, 0.79; *P* < 0.0001)		
			All-cause mortality	HR 0.66 (95% CI 0.54, 0.81; *P* < 0.0001)		
MERIT-HF Study Group. Lancet. 1999 (26)	Metoprolol	Placebo	All-cause mortality	RR 0.66 (95% CI 0.53, 0.81; *P* = 0.00009)	*N* = 3,991 patients chronic HF, NYHA class II-IV, EF ≤ 0.40	
			Death from worsening HF	RR 0.51 (95% CI 0.33, 0.79; *P* = 0.0023)		
MRA						
RALES Study Pitt B, et al. N Engl J Med. 1999 (27)	Spironolactone	Placebo	Death from any cause	RR 0.70 (95% CI 0.60, 0.82; *P* < 0.001)	*N* = 1,663 patients, HF, LVEF ≤ 35%, ACEi treatment, diuretic	Evidence showing benefit
			Death from cardiac causes	RR 0.69 (95% CI 0.58, 0.82; *P* < 0.001)		
			Hospitalization from cardiac causes	RR 0.70 (95% CI 0.59, 0.82; *P* < 0.001)		
			Combined death from cardiac causes or hospitalization from cardiac causes	RR 0.68 (95% CI 0.59, 0.78; *P* < 0.001)		
EMPHASIS-HF Zannad F, et al. N Engl J Med. 2011 (28)	Eplerenone	Placebo	Hospitalization for HF or death from cardiovascular causes	HR 0.63 (95% CI 0.54, 0.74; *P* < 0.001)	*N* = 2,737 patients, NYHA class II HF, EF≤35%	
			Hospitalization for HF	HR 0.58 (95% CI 0.47, 0.70 *P* < 0.001)		
SGLT2i						
DAPA-HF trial McMurray JJV, et al. N Engl J Med. 2019 (29)	Dapagliflozin	Placebo	Worsening HF (hospitalization or an urgent visit resulting in intravenous therapy) or CV death	HR 0.74 (95% CI 0.65, 0.85; *P* < 0.001)	*N* = 4,744 patients NYHA class II, III or IV HF, EF≤40%	Evidence showing benefit
			first worsening HF event	HR 0.70 (95% CI 0.59, 0.83)		
			Death from CV causes	HR, 0.82 (95% CI 0.69, 0.98)		
EMPEROR-Reduced trial Packer M, et al. N Engl J Med. 2021 (30)	Empagliflozin	Placebo	Death, hospitalization for HF or an emergent/urgent visit requiring intravenous treatment for HF	HR 0.76 (95% CI, 0.67–0.87; *P* < 0.0001)	*N* = 3,730 patients, NYHA class II to IV HF, EF≤40%	
			First and recurrent hospitalizations for HF	HR 0.70 (95% CI 0.58, 0.85; *P* < 0.001)		

ACEi, Angiotensin-converting enzyme inhibitor; ADHF, acute decompensated HF; ARNI, Angiotensin receptor-neprilysin inhibitor; cHF, Congestive heart failure; CI, Confidence interval; CV, Cardiovascular; EF, ejection fraction; HF, Heart failure; HR, Hazard ratio; MRA, Mineralocorticoid receptor antagonist; NT-proBNP, N-terminal pro B-type natriuretic peptide; NYHA. New York heart association; RR, Relative risk; SGLT2i, Sodium-glucose co-transporter 2 inhibitors.

ARB are used as alternative for HFrEF patients intolerant to ACEi. In the Val-HeFT trial, 5,010 patients were randomized to receive valsartan or placebo, with most on ACEi (93%), some on BB (35%), and few on spironolactone (5%), over a mean follow-up of 22.4 months (17). There was no significant difference in overall mortality (RR 1.02, *P* = 0.8), but a combined endpoint of mortality and morbidity showed a significant 13% reduction in the valsartan group (RR 0.87, *P* = 0.009), mainly due to fewer HF hospitalizations (*P* < 0.001) (17). In the CHARM Programme (18, 19), the CHARM-Alternative study (*N* = 2,028, median follow-up 33.7 months) showed candesartan significantly reduced cardiovascular death or HF hospitalization (HR 0.77, *P* = 0.0004), with cardiovascular death significant after adjustment (HR 0.80, *P* = 0.02) (19). The CHARM-Added study (*N* = 2,548, median follow-up 41 months) also found candesartan reduced cardiovascular death or HF hospitalization (HR 0.85, *P* = 0.011), with a significant reduction in cardiovascular deaths (adjusted *P* = 0.021) (18).

The PARADIGM-HF trial, a double-blind RCT, evaluated the effect of sacubitril/valsartan vs. enalapril added to recommended therapy on a composite endpoint of death from cardiovascular causes or hospitalization due to HF in 8,442 patients with HFrEF. Median follow-up was 27 months. Sacubitril/valsartan was superior to enalapril in reducing the risks of either cardiovascular death or HF-associated hospitalizations (*P* < 0.001), cardiovascular death (*P* < 0.001), and the risk of first hospitalization for HF (*P* < 0.001) (20). Another study examined the effect of sacubitril/valsartan on the risk of clinical progression compared to enalapril. Sacubitril/valsartan was associated with a 23% reduction in hospitalizations for worsening HF (*P* < 0.001). Sacubitril/valsartan also reduced the risk of other manifestations of disease progression, such as intensification of treatment, emergency room visits, cardiovascular-related hospitalizations, hospitalizations due to any cause, or need for intensive care. The 40% reduction in HF-associated hospitalization was significant within the first 30 days after randomization (21). A third study evaluated the time-averaged proportional change in N-terminal pro-B-type natriuretic peptide (NT-proBNP) concentration after 4 and 8 weeks of either sacubitril/valsartan or enalapril alone after hemodynamic stabilization in patients with HFrEF hospitalized due to acute decompensation. Sacubitril/valsartan therapy led to a greater reduction in the NT-proBNP concentration (*P* < 0.001) after 8 weeks of treatment (22). In the TRANSITION study, the reduction in NT-proBNP concentration was evaluated in patients with stabilized acute decompensated HFrEF who initiated sacubitril/valsartan either before or after discharge. Starting treatment in the hospital produced a greater reduction of NT-proBNP at discharge (23).

BB have also shown a clear benefit in terms of cardiovascular mortality and hospitalizations due to HF. In the CIBIS-II study, after 15 months of treatment, a 26% reduction in the risk of cardiovascular mortality (*P* = 0.0049) and a 31% reduction in hospital admission for worsening HF (*P* < 0.0001) were reported in the bisoprolol arm compared to placebo. All-cause mortality was also significantly lower in patients treated with bisoprolol (25). Another study comparing the effect of carvedilol to placebo showed a reduction in the mortality risk of 65% (*P* < 0.001) in the carvedilol group after 12 months. A 27% reduction in the risk of hospitalization due to cardiovascular causes (*P* = 0.036) and a 38% reduction in the risk of either hospitalization or death (*P* < 0.001) were also reported (24). A third double-blind randomized controlled study (MERIT-HF) enrolling almost 4,000 patients with chronic HFrEF showed that all-cause mortality was lower with metoprolol than with placebo (RR, 0.66, *P* = 0.00009) after 12 months of treatment; the risk of death from worsening HF was also lower with metoprolol (RR, 0.51, *P* = 0.0023) (26).

The risk of mortality and hospitalization has also been assessed in patients treated with MRAs. In an initial study of 1,663 patients (RALES) with severe HFrEF treated with ACEi, loop diuretics and digoxin showed a 30% reduction in death from any cause after the addition of spironolactone compared to placebo (*P* < 0.001) after 24 months. Death from cardiac causes was reduced by 21% in patients treated with spironolactone (*P* < 0.001), hospitalization from cardiac causes was reduced by 30% (*P* < 0.001), and the combination of both endpoints by 32% (*P* < 0.001) (27). In a second study with 2,737 patients with HFrEF treated with either eplerenone or placebo added to recommended therapy, the primary outcome was a composite of death from cardiovascular causes or hospitalization for HF: a reduction of 37% was observed in the intervention arm (HR, 0.63; 95% CI, 0.54–0.74; *P* < 0.001). The risk of death from any cause and the risk of hospitalization for HF were also significantly reduced (28).

SGLT2i have demonstrated a protective effect in patients with established HFrEF. The DAPA- HF study assessed the effect of dapagliflozin or placebo added to recommended therapy on the worsening of HF and cardiovascular death. A composite endpoint of worsening HF (including hospitalization or urgent visits requiring intravenous treatment) or cardiovascular death was significantly reduced in the dapagliflozin group (*P* < 0.001), as were each of those endpoints alone. The reduction in the primary event with dapagliflozin was rapidly statistically significant (28 days post-randomization), meaning that the benefit of the drug manifests promptly (29). Another study (EMPEROR-reduced trial) evaluated the efficacy of empagliflozin in patients with the same profile. Empagliflozin reduced by 24% the combined risk of death, hospitalization for HF or an emergent/urgent visit requiring intravenous treatment for HF (*P* < 0.0001). This benefit also reached early statistical significance (12 days after randomization) (30).

### Effects of pharmacological treatment for heart failure on sudden death

Results obtained on the effect of the different therapeutic options on SD are summarized in Table 2. There were no differences in the incidence of SD between enalapril and placebo groups in either the CONSENSUS trial or SOLVD (15, 16).

**Table 2:** Effect of pharmacological treatment on sudden death.

Publication	Intervention	Control	Endpoints	Results		N/demographic characteristics	Summary of the effect
ACEi							
CONSENSUS trial study Group. N Engl J Med. 1987 (15)	Enalapril	Placebo	Sudden cardiac death	Not significant		*N* = 253 patients, NYHA class IV cHF	Neutral/lack of evidence
SOLVD investigators. N Engl J Med. 1991 (16)	Enalapril	Placebo	Mortality due to an arrhythmia but not preceded by worsening congestive HF	Not significant		*N* = 2,569 patients < 80 years, NYHA class II or III cHF, EF ≤0.35	
ARB							
ELITE II Pitt B, et al. Lancet. 2000. (31)	Losartan	Captopril	Sudden death		Not significant	*N* = 3,152 patients ≥ 60 years, NYHA class II-IV HF and EF ≤ 40%	Poor or inconsistent evidence
CHARM program Solomon SD, et al. Circulation. 2004 (32)	Candesartan	Placebo	Sudden death		Numerical occurrence of SD: • lower in the candesartan group versus placebo in both CHARM-Alternative (80 [7.9%] versus 111 [10.9%]) and CHARM-Added (150 [11.8%] versus 168 [13.2%]), but no definitive conclusions	*N* = 2028 patients LVEF ≤ 40% and ACE intolerant (CHARM-alternative) *N* = 2,548 patients LVEF ≤ 40% and already on ACEi (CHARM-Added) *N* = 3023 patients LVEF ≤ 40% (CHARM-Preserved)	
ARNI							
PARADIGM-HF Desai AS, et al. Eur Heart J. 2015 (33)	Sacubitril/valsartan	Enalapril	Death due to worsening HF		HR 0.79 (95% CI 0.64, 98; *P* = 0.034)	*N* = 8,399 patients chronic HF, NYHA class II-IV, LVEF ≤ 40%	Evidence showing benefit
			Death due to sudden death		HR 0.80 (95% CI 0.68, 0.94; *P* = 0.008)		
Beta-blockers							
Packer M, et al. N Engl J Med. 1996 (24)	Carvedilol	Placebo	Sudden death	1.7% versus 3.8%		*N* = 1,094 patients, chronic HF, LVEF ≤ 0.35	Evidence showing benefit
CIBIS-II Investigators and Committees. Lancet. 1999 (25)	Bisoprolol	Placebo	Sudden death	HR 0.56 (95% CI 0.39, 0.80; *P* = 0.0011)		*N* = 2,647 patients, NYHA class III or IV, LVEF ≤ 35%, receiving diuretics and ACEi	
MERIT-HF Study Group. Lancet. 1999 (26)	Metoprolol	Placebo	Sudden death	HR 0.59 (95% CI 0.45, 0.78; *P* = 0.0002)		*N* = 3,991 patients chronic HF, NYHA class II-IV, EF ≤ 0.40	
MRA							
RALES Study Pitt B, et al. N Engl J Med. 1999 (27)	Spironolactone	Placebo	Sudden death	HR 0.71 (95% CI 0.60, 0.82; *P* = 0.02)		*N* = 1,663 patients, HF, LVEF ≤ 35%, ACEi treatment, diuretic	Evidence showing benefit
EMPHASIS-HF Zannad F, et al. N Engl J Med. 2011 (28)	Eplerenone	Placebo	Sudden cardiac death	HR 0.76 (95% CI 0.54, 1.07; *P* = 0.12)		*N* = 2,737 patients, NYHA class II HF, EF ≤ 35%	
Bapoje SR, et al. Circ Heart Fail. 2013 (34)	MRAs	Several control arms	Sudden cardiac death	OR 0.77 (95% CI 0.66, 0.89; *P* = 0.001)		*N* = 11,875 patients LVEF ≤ 45%	
SGLT2i							
*Post hoc* analysis of DAPA-HF Curtain JP, et al. Eur Heart J. 2021 (35)	Dapagliflozin	Placebo	Serious ventricular arrhythmia, resuscitated cardiac arrest, or sudden death	HR 0.79 (95% CI 0.63, 0.99; *P* = 0.037)		*N* = 4,744 patients ≥ 18 years, NYHA class II-IV, LVEF ≤ 40%	Neutral/lack of evidence
			Sudden death	HR 0.81 (95% CI 0.62, 1.07)			

ACEi, angiotensin-converting enzyme inhibitor; ARNI, angiotensin receptor-neprilysin inhibitor; cHF, congestive heart failure; EF, ejection fraction; HF, hearth failure; HR, hazard ratio; LVEF, left ventricular ejection fraction; MRA, mineralocorticoid receptor antagonist; NYHA, New York Hearth Association; SGLT2i, sodium-glucose co-transporter 2 inhibitors.

The ELITE II trial, involving 3,152 patients over 60 years, found no significant differences between losartan and captopril in SD (HR 1.13, *p* = 0.16) or resuscitated arrests (HR 1.25, *p* = 0.08) (31). The CHARM program lacked the power to definitively assess candesartan's impact on causes of death. Although SD rates were numerically lower with candesartan in CHARM-Alternative (7.9% vs. 10.9%) and CHARM-Added (11.8% vs. 13.2%), no definitive conclusions were made (32).

The clinical evidence on ARNI shows a clear benefit in the reduction of the incidence of SD in patients with HFrEF. Mode of death was adjudicated by a blinded clinical endpoints committee and, at the end of a 42-month follow-up period (mean follow-up was 27 months), the risk of SD was reduced by 20% (*P* = 0.008) while the risk of death from worsening HF was also reduced by 21% (*P* = 0.034) (33).

Several studies have addressed the incidence of SD in patients with HFrEF treated with BB (36). The CIBIS-II study reported a 44% reduction in the risk of SD (*P* = 0.0011) with bisoprolol compared to placebo (25). In the MERIT-HF study, there was a 41% reduction in SD (*P* = 0.0002) in the metoprolol group compared to the placebo group (26). Results obtained with carvedilol were similar and a 55% reduction was reported (24).

An overall benefit for MRA has been observed in the reduction of SD. In the RALES study, a 29% reduction in the relative risk of SD (*P* = 0.02) was described in the arm treated with spironolactone compared to the arm treated with placebo (27). In contrast, in the EMPHASIS-HF study, the reduction in the relative risk of SD of 24% with eplerenone vs. placebo was not statistically significant (*P* = 0.12) (28). A subsequent metaanalysis evaluated the relative risk reduction of SD with any MRA compared to control arms. Eight randomized controled trials that included almost 12,000 patients met the inclusion criteria; five examined the role of spironolactone, two eplerenone, and one canrenone. MRAs were associated with a 23% reduction in sudden cardiac death compared to controls (*P* = 0.001). Similar reductions were observed in cardiovascular and total mortality (34).

The effect of SGLT2i on SD was examined in a *post hoc* analysis of the DAPA-HF study. Dapagliflozin significantly reduced a composite endpoint of serious ventricular arrhythmia, resuscitated cardiac arrest, and SD compared to placebo, but the effect on SD alone was not significant (35).

### Effects of pharmacological treatment for heart failure on reverse cardiac remodeling

Some differences have been shown in the effect of the different therapeutic options on reverse cardiac remodeling (Table 3). A substudy from the SOLVD study was carried out to elucidate whether enalapril inhibited remodeling in the patients enrolled. A pool of 301 patients from 5 centers were examined using Doppler echocardiography before randomization and after 4 and 12 months of therapy with either enalapril or placebo. EF remained unchanged in both arms after 12 months (37). The CARMEN study evaluated the suitability of the combination of ACEi and BB for the treatment of chronic HFrEF, as well as the recommendation to initiate treatment with ACEi and only add BB in symptomatic patients. Regarding reverse cardiac remodeling, in patients treated with enalapril, changes in LVEF were not significant at 6 and 12 months and were statistically greater at 18 months (38).

**Table 3:** Effect of pharmacological treatment on reverse cardiac remodeling.

Publication	Intervention	Control	Endpoints	Results	N/Demographic characteristics	Summary of the effect
ACEi						
Greenberg B, et al. Circulation. 1995 (37)	Enalapril	Placebo	EF		*N* = 301 patients from SOLVD study, 21–80 years, LVEF≤0.35	Neutral/lack of evidence
CARMEN Remme WJ, et al. Cardiovasc Drugs Ther. 2004 (38)	Enalapril	-	Mean change of LVEF from baseline	Not significant at month 6 and 12 Significant increase at month 18 (*P* < 0.05)°	*N* = 572 mild HF patients	
ARB						
Val-HeFT echocardiographic study Wong, et al. J Am Coll Cardiol. 2002 (39)	Valsartan	Placebo	LVDD LVEF	Decrease in indexed LVDD compared to placebo (−0.12 ± 0.4 versus −0.05 ± 0.4 cm/m²; *p* < 0.00001) and an increase in LVEF (+4.5 ± 8.9% versus +3.2 ± 8.6%; *p* < 0.00001)	*N* = 5,010 patients, NYHA class II-IV HF, taking ACEi and/or BB	Poor or inconsistent evidence
RESOLVD pilot study McKelvie RS, et al. Circulation. 1999 (40)	Candesartan	Enalapril versus combination of candesartan and enalapril	LVEDV and LVESV LVEF	Smaller increase in LVEDV and LVESV (EDV 8 ± 4 ml; ESV 1 ± 4 ml; *p* < 0.01) versus to candesartan alone (EDV 27 ± 4 ml; ESV 18 ± 3 ml) or enalapril alone (EDV 23 ± 7 ml; ESV 14 ± 6 ml). No significant differences in LVEF were observed among the groups	*N* = 768 patients, NYHA class II-IV HF, EF < 0.40 and 6MWD <500 m	
HEAVEN Willenheimer R, et al. Int J Cardiol. 2002 (41)	Valsartan	Enalapril	LVEDD	Statistically significant decrease in LVEDD only in the valsartan group (mean change −4.1 mm/m²; 95% CI −2.17 to −5.98; *p* < 0.001)	*N* = 141 patients, mean 68 years, 74% males, stable mild/moderate HF and LVEF ≤ 0.45	
Lang RM, et al. J Am Coll Cardiol. 1997 (42)	Losartan	Enalapril	LVEF	No significant changes in LVEF among the treatment groups (*p* = 0.75); a statistically significant improvement of 2.3% in LVEF observed from baseline to week 12 in the 50 mg losartan group	*N* = 116 patients, NYHA class II-IV cHF, LVEF ≤ 0.45, treated with ACEi and diuretic agents	
Kasama S, et al. Heart. 2006 (43)	Valsartan	Enalapril	LVEDV LVEF	In the valsartan group: • Significant decrease in LVEDV (from 172 to 152 ml; *p* < 0.05) Significant increase in LVEF (from 31% to 39%; *p* < 0.001)	*N* = 50 patients cHF, LVEF < 40%	
ARNI						
Januzzi JL, et al. JAMA. 2019 (44)	Sacubitril/valsartan	None	Mean LVEF change (%)	5.2% (95% CI 4.8%, 5.6%) at 6 months (*P* < 0.001) 9.4% (95% CI 8.8%, 9.9%), at 12 months (*P* < 0.001)	*N* = 794 patients with HFrEF, mean 65.1 years, 28.5% female, mean LVEF = 28.2%)	Evidence showing benefit
Beta-blockers						
CARMEN Remme WJ, et al. Cardiovasc Drugs Ther. 2004 (38)	Carvedilol	–	Mean change of LVEF from baseline	Significant increase at month 6, 12 (*P* < 0.001) and 18 (*P* < 0.01)	*N* = 572 mild HF patients	Evidence showing benefit
Colucci WS, et al. Circulation. 2007 (45)	Metoprolol 200 mg Metoprolol 50 mg	Placebo	LVEF change (%) at 6 months	Placebo: LSM change 1.2 (95% CI −1.1, 3.6; *P* = 0.31) 50 mg: LSM change 2.9 (95% CI 0.4, 5.5; *P* = 0.022) 200 mg: LSM change 5.6 (95% CI 3.0, 8.1; *P* < 0.001)	*N* = 149 patients NYHA class I, LVEF < 40%	
			LVEF change (%) at 12 months	Placebo: LSM change 0.0 (95% CI −2.5, 2.5; *P* = 0.99) 50 mg: LSM change 3.9 (95% CI 1.4, 6.5; *P* = 0.003) 200 mg: LSM change 6.2 (95% CI 3.6, 8.7; *P* < 0.001)		
MRA						
Vizzardi E, et al. Am J Cardiol. 2010 (46)	Spironolactone	Placebo	LVEF change (%) at 6 months	Placebo: change from 35.2 ± 4.1 to 34.9 ± 8.1 (*P* = 0.59) Spironolactone: change from 34.6 ± 9.8 to 38.4 ± 6.3 (*P* = 0.001)	*N* = 168 patients NYHA class III-IV HF, LVEF ≤ 40%	Poor or inconsistent evidence
Udelson JE, et al. Circ Heart Fail. 2010 (47)	Eplerenone	Placebo	LVEF increase (%) at 36 months	Placebo: 1.4 (0.41) Eplerenone: 1.8 (0.37) (*P* = 0.47)	*N* = 226 patients, NYHA class II/III HF, LV systolic dysfunction, LVEF ≤ 35%	
SGLT2i						
Singh JSS, et al. Diabetes Care. 2020 (48)	Dapagliflozin	Placebo	LVEF change (%)	0.69 (95% CI −3.32, 4.69; *P* = 0.732)	*N* = 56 patients type 2 DM, LV systolic dysfunction	Poor or inconsistent evidence
Santos-Gallego C, et al. JACC. 2021 (49)	Empagliflozin	Placebo	Absolute LVEF change from baseline	6 ± 4.2 versus −0.1 ± 3.9 (*P* < 0.001)	*N* = 84 nondiabetic HFrEF patients	
DAPA-MODA trial Pascual-Figal DA, et al. Eur J Heart Fail. 2023 (50)	Dapagliflozin	–	LVEF change (%) at 6 months	4,9% (95% CI 0.2%, 9.9%; *P* = 0.040)	*N* = 162 patients with stable chronic HF, 64.2% male, 70.5 ± 10.6 years, 52% LVEF > 40%	

ACEi, angiotensin-converting enzyme inhibitor; ARNI, angiotensin receptor-neprilysin inhibitor; BB, beta-blocker; cHF, congestive heart failure; DM, diabetes mellitus; EF, ejection fraction; HF, heart failure; HFrEF, heart failure reduced ejection fraction; LVEF, Left ventricular ejection fraction; LSM, Least square mean; MRA, mineralocorticoid receptor antagonist; 6MWD, 6-minute walk distance; NYHA, New York Heart Association; SGLT2i, sodium-glucose co-transporter 2 inhibitors.

The Val-HeFT substudy found valsartan significantly reduced LVDD (−0.12 vs. −0.05 cm/m^2^; *p* < 0.00001) and increased LVEF (+4.5% vs. + 3.2%; *p* < 0.00001) at 18 months (39). In the RESOLVD pilot, combination therapy (candersatan plus enalapril) led to smaller increases in LVEDV (8 vs. 27 ml) and LVESV (1 vs. 18 ml) than candesartan alone (40). An open-label study with 445 HFrEF patients showed similar LVEF, LVSD, and LVDD improvements for valsartan and enalapril (51). The HEAVEN study found a significant LVEDD decrease only with valsartan (−4.1 mm/m^2^; *p* < 0.001) (41). Another trial with 116 patients replacing ACEi with losartan showed a 2.3% LVEF improvement with high-dose losartan (50 mg) at week 12 (42). A trial with 50 HFrEF patients reported significant decreases in LVEDV (172 to 152 ml, *p* < 0.05) and increases in LVEF (31% to 39%; *p* < 0.001) only with valsartan (43).

Clinical evidence with ARNI has demonstrated also a clear benefit in terms of reverse cardiac remodeling. The PROVE-HF study enrolled 794 patients with HFrEF who were switched from ACEi/ARB to ARNI therapy. At 6 months, left ventricle and left atrial volume indexes, E/e’ ratio, LV mass index and LVEF were significantly improved compared to baseline. After 12 months, sacubitril/valsartan produced a mean LVEF increase of 9.4%. Moreover, 25% of patients experienced a LVEF increase of 13.4% or more. Patients with new-onset HF or who were ACEi/ARB naive at baseline showed mean improvements in LVEF of 12.8% (44).

In the aforementioned CARMEN study, patients receiving carvedilol monotherapy showed a significant increase in LVEF at 6, 12 and 18 months (38). The REVERT study elucidated the effect of therapy with the BB metaprolol on left ventricular remodeling in asymptomatic patients with left ventricular systolic dysfunction. A total of 149 patients were randomized to three treatment groups (200 mg, 50 mg, and placebo). LVEF increased significantly in patients treated with metoprolol after 6 months compared to placebo. After 12 months, LVEF remained unchanged in the placebo group, but increased significantly by 3.9% in patients receiving 50 mg metoprolol and by 6.2% in patients receiving 200 mg metoprolol. Changes were similar at 6 months (45).

A randomized control trial examined the effect of spironolactone on LV function and the functional capacity of patients with mild to moderate HF. LVEF increased from 35.2% to 39.1% ± 3.5% after 6 months of spironolactone treatment, while the placebo arm showed no significant differences (*P* = 0.003) (46). A second study evaluated the effect of eplerenone added to contemporary background therapy on ventricular remodeling in patients with HFrEF. After 36 weeks of treatment, no effects were observed in left heart remodeling parameters with eplerenone compared to placebo (47). Thus, the clinical evidence obtained with MRA in reverse cardiac remodeling is limited and inconsistent.

In the case of SGLT2i, the sample size of the studies addressing reverse cardiac remodeling to date is limited. The REFORM study only included 56 patients assigned to either dapagliflozin or placebo and no statistically significant effect was observed on LVEF changes at 12 months (48). Another study evaluating the role of empagliflozin on LV function and volumes in 84 nondiabetic HFrEF patients showed a statistically significant improvement in LV volumes, LV mass and LVEF at 6 months of treatment (49). Recently, the DAPA-MODA, a single-arm study including 162 patients (48% LVEF ≤40%, 52% LVEF >40%) showed a favorable and significant impact of dapagliflozin in both atrial and ventricular remodeling parameters including an increase of 5% in LVEF (*p* = 0.040), assessed by echocardiography and interpreted in a blinded manner (50).

### Effects of pharmacological treatment for heart failure on renal protection

HF is associated with a greater decline in renal function over time. However, not all available HF treatments exert a positive effect on renal function (Table 4). In the SOLVD study, more patients receiving enalapril showed a serum creatinine increase of at least 2 mg/dl or a potassium increase of at least 5.5 mmol/L than in the placebo arm (*P* < 0.01) (16). In the CONSENSUS trial, however, the increase in serum creatinine levels did not lead to significant differences in treatment discontinuation between the enalapril and placebo arms (15). Clinical evidence suggests a beneficial clinical effect even in patients with HFrEF who suffer a decline in renal function after initiating treatment with ACEi (57). However, there is no clinical evidence to show that ACEi reduces the risk of lower eGFR. Similar data are available for ARBs (58). In addition, baseline chronic kidney disease (CKD) should not preclude ACEi/ARB use, as their efficacy persists in CKD, supported by trials like CONSENSUS, SOLVD, ValHeFt, and CHARM (59). However, specific studies on RAAS inhibitors in advanced CKD (GFR <15 ml/min/1.73 m^2^) are limited, and safety should be confirmed in long-term observational studies. KDIGO guidelines recommend ACEi and ARBs in CKD stage 5, with moderate evidence for use in dialysis and weak evidence for non-dialysis patients (60).

**Table 4:** Effect of pharmacological treatment on renal protection.

Publication	Intervention	Control	Endpoints	Results	N/demographic characteristics	Summary of the effect
ACEi						
CONSENSUS trial study Group. N Engl J Med. 1987 (15)	Enalapril	Placebo	Discontinuation due to serum creatinine increase	4/126 patients treated with placebo versus 6/127 patients treated with enalapril	*N* = 253 patients, NYHA class IV cHF	Neutral/lack of evidence
SOLVD investigators. N Engl J Med. 1991 (16)	Enalapril	Placebo	Difference on serum creatinine levels	0.2 mmol/L	*N* = 2,569 patients < 80 years, NYHA class II or III cHF, EF ≤0.35	
			Difference on potassium levels	88 mmol/L		
			Patients with creatinine increase > 177 µmol/L	10.7% versus 7.7% (*P* < 0.01)		
			Patients with potassium increase > 5.5 mmol/L	6.4% versus 2.5% (*P* < 0.01)		
ARNI						
PARADIGM-HF McMurray J, et al. N Engl J Med. 2014 (20)	Sacubitril/valsartan	Enalapril	Elevated serum creatinine (≥2.5 mg/dl)	3.3% versus 4.5% (*P* = 0.007)		Evidence showing benefit
			Elevated serum creatinine (≥6 mmol/L)	4.3% versus 5.6% (*P* = 0.007)	*N* = 8,442 patients, class II, III or IV HF, EF ≤ 40%	
PARADIGM-HF analysis Damman K, et al. JACC Heart Fail. 2018 (52)	Sacubitril/valsartan	Enalapril	Change in eGFR at month 48	HR 0.63 (95% CI 0.42, 0.95; *P* = 0.028)	*N* = 8,339 patients, HFrEF	
	Sacubitril/valsartan with CDK	Sacubitril/valsartan without CDK	CV death and hospitalization for HF composite	HR 0.79 (95% CI 0.69, 0.90) versus HR 0.81 (95% CI 0.73–0.91) (*P* = 0.70)		
Secondary analysis PARADIGM-HF trial Packer M, et al. Lancet Diabetes Endocrinol. 2018 (53)	Sacubitril/valsartan	Enalapril	Decline in eGFR	–1.3 versus –1.8 ml/min per 1.73 m² per year (*P* < 0.0001)	*N* = 8,339 patients, HFrEF	
			Difference in eGFR decline with ARNI and ACEi	With type 2 DM: 0.6 ml/min per 1.73 m² per year (95% CI 0.4, 0.8) Without type 2 DM: 0.3 ml/min per 1.73 m² per year (95% CI 0.2, 0.5) (*P* = 0.038)		
PARADIGM-HF secondary analysis Desai AS, et al. JAMA Cardiol. 2017 (54)	Sacubitril/valsartan	Enalapril	Severe hyperkalemia in patients receiving MRA at baseline	HR 1.37 (95% CI 1.06, 1.76; *P* = 0.02)	*N* = 8,339 patients, HFrEF	
Beta-blockers						
Packer M, et al. N Engl J Med. 1996 (24)	Carvedilol	Placebo	Renal adverse reactions	Not reported	*N* = 1,904 patients, chronic HF, LVEF ≤ 0.35	Neutral/lack of evidence
COPERNICUS Study Packer M, et al. Circulation. 2002 (55)	Carvedilol	Placebo	Serious renal adverse events	Not reported	*N* = 2,289 patients HF, EF < 25%	
MRA						
RALES Study Pitt B, et al. N Engl J Med. 1999 (27)	Spironolactone	Placebo	Median creatinine concentration	Placebo: no changes Spironolactone: 4–9 µmol/L (*P* < 0.001)	*N* = 1,663 patients, HF, LVEF≤35%, ACEi treatment, diuretic	Neutral/lack of evidence
			Median Potassium concentration	Placebo: no changes Spironolactone: increase of 0.30 mmol/L (*P* < 0.001)		
EMPHASIS-HF Zannad F, et al. N Engl J Med. 2011 (28)	Eplerenone	Placebo	Change in creatinine concentration	At 1 month: • Placebo: 6.2 ± 25.6 µmol/L versus • Eplerenone: 13.3 ± 30.9 µmol/L At cutoff: • Placebo: 3.5 ± 35.4 µmol/L versus • Eplerenone: 8.0 ± 32.7 µmol/L	*N* = 2,737 patients, NYHA class II HF, EF≤35%	
			Change in Potassium concentration	At 1 month: • Placebo: 0.04 ± 1.16 mmol/L versus • Eplerenone: 0.16 ± 0.51 mmol/L (*P* = 0.001) At cutoff: • Placebo: 0.05 ± 0.53 mmol/L versus • Eplerenone: 0.16 ± 0.56/L (*P* = 0.001)		
SGLT2i						
DAPA-HF trial McMurray JJV, et al. N Engl J Med. 2019 (29)	Dapagliflozin	Placebo	% of worsening renal function	HR 0.71 (CI 95% 0.44, 1.16; NA)	*N* = 4,744 patients NYHA class II, III or IV HF, EF≤40%	Evidence showing benefit
			Creatinine levels	HR 2.41 (CI 95% 2.21, 2.62; *P* < 0.001)		
EMPEROR-Reduced trial Packer M, et al. N Engl J Med. 2021 (30)	Empagliflozin	Placebo	Composite renal outcome	HR 0.50 (CI 95% 0.32, 0.77)	*N* = 3,730 patients, NYHA class II to IV HF, EF ≤ 40%	
			Mean slope of change in eGFR (ml/min/1.73 m^2)^	HR 1.73 (CI 95% 1.10, 2.37; *P* < 0.001)		
DAPA-HF Jhund PS, et al. Circulation. 2021 (56)	Dapagliflozin	Placebo	≥50% sustained decline eGFR or end-stage renal disease or renal death	HR 0.71 (CI 95% 0.44–1.16; *P* = 0.17)	*N* = 4,742 patients HFrEF ± type 2 DM, eGFR ≥ 30 mlmin^−1^·1,73 m^−2^	
			Change in eGFR from baseline to day 14	−4.19 (95% CI −4.52, −3.87) versus −1.09 (95% CI −1.42, −0.77) (*P* < 0.001)		
			Change in eGFR from day 14 to 720	−1.09 (95% CI −1.40, −0.77) versus −2.85 (95% CI −3.17, −2.53) (*P* < 0.001)		

ACEi, angiotensin-converting enzyme inhibitor; ARNI, angiotensin receptor-neprilysin inhibitor; CI, confidence interval; CV, cardiovascular; DM, diabetes mellitus; eGFR, estimated glomerular filtration rate; HF, heart failure; HFrEF, heart failure reduced ejection fraction; HR, hazard ratio; MRA, mineralocorticoid receptor antagonist; SGLT2i, sodium-glucose co-transporter 2 inhibitors.

In the PARADIGM-HF trial, adverse events due to serum creatinine levels ≥ 2.5 mg/dl and serum potassium levels > 6.0 mmol/L were less frequent with sacubitril/valsartan than with enalapril (*P* < 0.05 for both comparisons) (20). A comprehensive analysis of the PARADIGM-HF trial illustrated a clear 37% reduction in renal function decline among patients treated with sacubitril/valsartan compared to patients treated with enalapril. Additionally, sacubitril/valsartan showed a consistent clinical benefit for cardiovascular death and hospitalization in the HF composite outcome, irrespective of the presence or absence of chronic kidney disease (52). Another study assessed differences in the effect of sacubitril/valsartan on renal function in patients with type 2 diabetes mellitus (T2DM). Patients treated with sacubitril/valsartan showed a slower decline in eGFR than those treated with enalapril. The difference between the sacubitril/valsartan and the enalapril arms observed in patients with T2DM was twice as high as that observed in patients without T2DM (53). A subsequent secondary analysis of the PARADIGM-HF trial described how sacubitril/valsartan reduced the risk of severe hyperkalemia compared to enalapril in patients treated with MRA at baseline (54).

Clinical evidence on the effect of BB on renal function is limited. Their use does not cause renal function to worsen over time, as is the case with ACEi. Several studies have reported that patients in the lowest GFR strata show a greater relative risk reduction with BB (57, 61, 62). No adverse events related to the worsening of renal function were reported in the randomized clinical trials addressing the effect of carvedilol on mortality and morbidity in patients with HFrEF (24, 55).

MRA does not seem to exert a protective effect on renal function. In fact, spironolactone was associated with significant but not clinically relevant increases in creatinine and potassium (27). In EMPHASIS study, eplerenone was associated with a significant but not clinically relevant serum potassium increase at one month and at the cutoff date, and with a non-significant increase in serum creatinine (28). In the EPHESUS study, eplerenone produced a decline in eGFR compared with placebo (*P* < 0.0001) that appeared within the first month and lasted throughout the 24-month follow-up (63). Thus, the initiation of treatment with MRA produces an acute decline in eGFR that is maintained throughout administration (57).

Two studies have corroborated the renal protective effect of SGLT2i. In the DAPA-HF trial, the percentage of patients with worsening renal function was similar in the dapagliflozin and placebo arms (29). In a *post hoc* analysis of the same study, a composite renal outcome (≥50% sustained decline eGFR or end-stage renal disease or renal death) was not reduced by dapagliflozin, although the rate of decline in eGFR between day 14 and 720 was less with dapagliflozin than with placebo (56). In the EMPEROR-REDUCED trial, a composite renal outcome was assessed in patients treated with either empagliflozin or placebo, and a statistically significant difference emerged in favor of empagliflozin (30). Both studies demonstrated a reduction in the risk of worsening renal function.

### Effects of pharmacological treatment for heart failure on hospitalized patients

The effect of the different therapeutic options on hospitalized patients is summarized in Table 5. To date, no robust data are available from randomized controlled trials on the initiation of ACEi in hospitalized patients (71). The CONSENSUS study, for instance, only included 253 hospitalized patients from a total of 1,987 recruited patients (15).

**Table 5:** Effect of pharmacological treatment in hospitalized patients.

Publication	Intervention	Control	Endpoints	Results	N/demographic characteristics	Summary of the effect
ACEi						
Not studied						Poor or inconsistent evidence
ARB						
GWTG-HF Registry Assessment from a Get With the Guidelines-Heart Failure (GWTG-HF) registry from February 2009 through March 2010 (64)					*N* = 9,474 patients	Poor or inconsistent evidence
ARNI						
PIONEER-HF Study subanalysis Velazquez EJ, *et al*. Late Breaker AHA 2018. Chicago, IL, USA (65)	Sacubitril/valsartan	Enalapril	Combined risk of sdeath, rehospitalization for HF, need of LV assistance or inclusion on cardiac transplant waiting list	HR 0.54 (95% IC 0.37, 0.79; *P* = 0.001)		Evidence showing benefit
PIONEER-HF analysis Ambrosy AP, et al. J Am Coll Cardiol. 2020 (66)	*de novo* HF	Worsening chronic HF	Composite of CV death or rehospitalization for HF	Significant improvement (*P* < 0.0001)	*N* = 881 patients HF, EF ≤ 40%	
Beta-blockers						
IMPACT-HF trial Gattis WA, et al. J Am Coll Cardiol. 2004 (67)	Predischarge carvedilol	Physician discretion post-discharge initiation	Death + rehospitalization composite endpoint	84 (45.4) versus 82 (46.1) (95% CI −0.0959, 0.1091)	*N* = 363 patients hospitalized for HF	Poor or inconsistent evidence
Prins KW, et al. JACC Heart Fail. 2015 (68)	Continuing beta-blocker therapy	Discontinuing beta-blocker therapy	Risk of in-hospital mortality	RR 3.72 (95% CI 1.51, 9.14)	Meta-analysis *N* = 2,704 patients ADHF on BB	
			Short-term mortality	RR 1.61 (95% CI 1.04, 2.49)		
			Combined short-term rehospitalization or death	RR 1.59 (95% CI 1.03, 2.45)		
MRA						
Rossi R, et al. J Renin Angiotensin Aldosterone Syst. 2015 (69)	Initiating MRAs at discharge	Initiating MRAs at 30–90 days after discharge	Mortality at six months	HR 1.72 (95% CI 0.96, 2.84)	*N* = 685 patients, decompensated congestive HF	Neutral/lack of evidence
			Mortality at one year	HR 1.93 (95% CI 1.18, 3.14)		
SGLT2i						
EMPULSE Voors AA, et al. Nat Med. 2022 (70)	Empagliflozin	Placebo	Death from any cause, number of HF events and time to first HF event, or a 5 point or greater difference in change from baseline in the KCCQ-TSS at 90 days.	Win ratio 1.36 (95% CI 1.09, 1.68; *P* = 0.0054)	*N* = 530 patients, acute *de novo* or decompensated chronic HF	Evidence showing benefit

ACEi, angiotensin-converting enzyme inhibitor; ADHF, acute decompensated heart failure; ARNI, angiotensin receptor-neprilysin inhibitor; BB, beta-blocker; CI, Confidence interval; CV, cardiovascular; HF, heart failure; HR, hazard ratio; KCCQ-TSS, Kansas City cardiomyopathy questionnaire total symptom score; MRA, mineralocorticoid receptor antagonist; RR, relative risk; SGLT2i, sodium-glucose co-transporter 2 inhibitors.

An analysis of the GWTG-HF registry revealed that hospitalized patients who continued ACEi/ARB therapy experienced significantly lower mortality rates at 30 days, 90 days, and 1 year, as well as reduced 30-day readmission rates, compared to those who discontinued ACEi/ARB therapy (64).

ARNI have shown clinical benefit in hospitalized patients. In a subanalysis of the PIONEER-HF study, a 46% reduction was recorded in the combined risk of death, readmission for HF, need for LV assistance or inclusion in a heart transplantation waiting list (65). In another study (PIONEER-HF), patients with *de novo* HF had lower risk of either cardiovascular death or rehospitalization for HF than patients with previous history of HF. Likewise, patients not treated with ACEi or angiotensin receptor blockers at admission also showed a significantly lower incidence of the same composite endpoint (66).

The IMPACT-HF study, a prospective trial in 363 patients, showed a non-significant trend towards a benefit in terms of a lower composite of death or rehospitalization for the predischarge initiation of carvedilol in stabilized patients compared to initiation of any BB more than two weeks after discharge (67). A meta-analysis that included five observational studies and one randomized clinical trial showed that the discontinuation of BB in patients admitted to hospital was associated with increased in-hospital and short-term mortality (68).

No randomized clinical trials have addressed the role of MRA in hospitalized patients and available evidence is currently limited to a single-center observational study of 685 patients discharged after admission for acute HF. In this study, starting MRA in the hospital was associated with significantly lower mortality than late initiation (69). In contrast, the EMPULSE trial (empagliflozin) showed a clear clinical benefit in the composite endpoint (death from any cause, number of HF events and time to first HF event, or a 5-point or greater change from baseline in the Kansas City Cardiomyopathy Questionnaire Total Symptom Score at 90 days) (70).

## Discussion

This review highlights that the current guideline-directed medications (ACEi/ARB/ARNi + BB + MRA + SGLT2i) have a positive effect on the main components of disease progression in HFrEF patients. However, the extent of protection varies among these drugs (Graphical Abstract). All medications prevent cardiovascular mortality and HF hospitalization (15–30), but ACEi lack clear evidence for SD, renal protection, or benefits in hospitalized patients, possible due to older studies (15, 16, 71). ARNI (sacubitril-valsartan) extends benefits beyond ACEi/ARB, showing improvements in all disease progression components, and is increasingly recommended over ACEi (20–23, 33, 44, 52–54, 65, 66).

The benefit of BB is clear for decades, with numerous trials supporting the effect on cardiac protection and the reduction of related complications, with a neutral effect in renal function (24, 25, 55, 57, 61, 62). MRAs are limited by the risk of hyperkalemia in kidney disease (27). Finally, SGTL2i, the last pillar incorporated to HFrEF, significantly reduce the risk of HF decompensation and improve renal protection, with less evidence on cardiac remodeling and SD (29, 30, 35, 48–50, 56).

**Figure 1:**
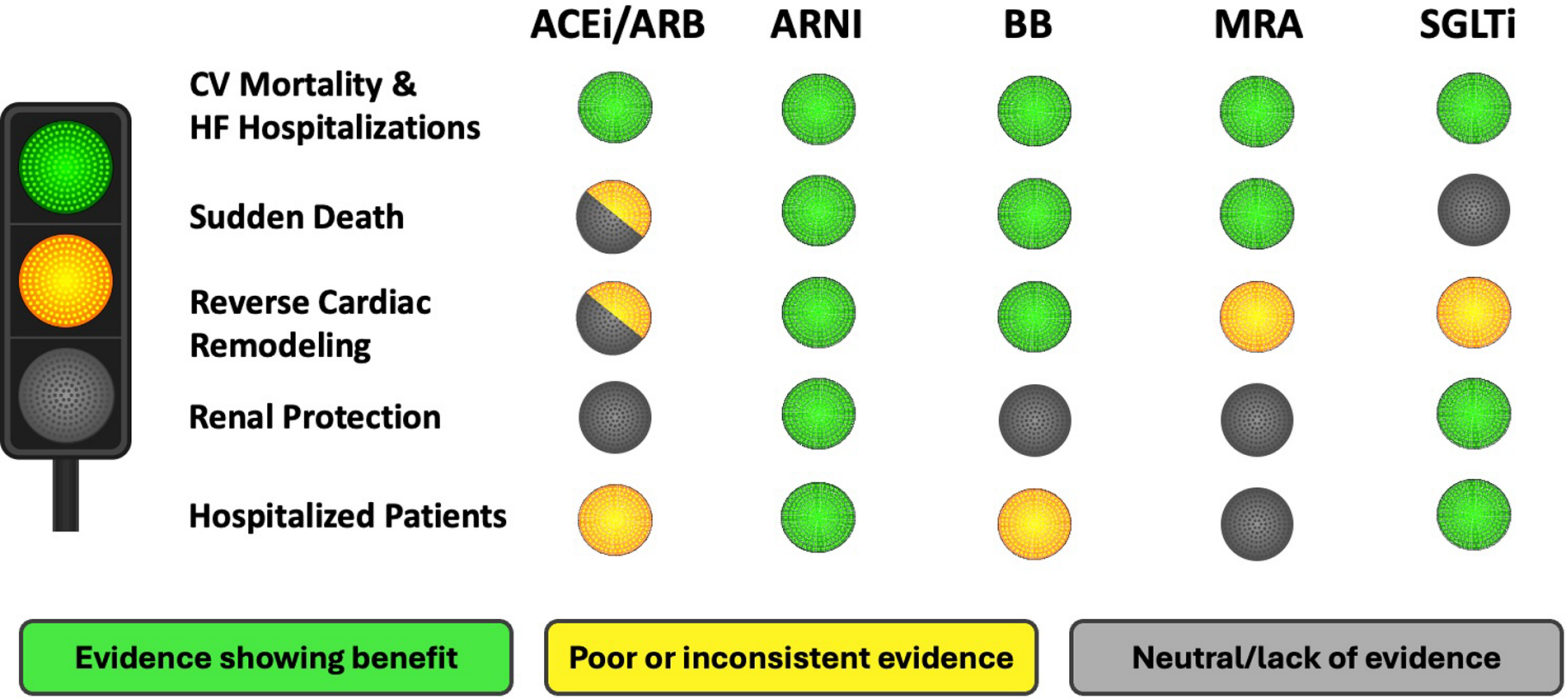
Suitability of therapeutic options for the treatment of HFrEF. HFrEF, heart failure with reduced ejection fraction; CV, cardiovascular; ACEi, angiotensin-converting enzyme inhibitor; ARB, angiotensin II receptor blocker; ARNI, angiotensin receptor-neprilysin inhibitor; BB, beta-blockers; MRA, mineralocorticoid receptor antagonist; SGLT2i, sodium-glucose co-transporter 2 inhibitor.

In conclusion, all currently recommended medications for HFrEF have a positive effect on mortality and hospitalization rates. Considering the relevance of preventing SD as mode of death, ARNI, BB, and MRA are particularly effective. In addition, organ protection should be considered. In this regard, ARNI, BB, and SGLT2i improve LVEF and reverse parameters of cardiac remodeling, with ARNI and SGLT2i also offering renal protection. Likewise, it is important to consider effective and safe medications within the early phase of HF hospitalizations, and ARNI and SGLT2i are the main options in this acute setting (Figure 1). Therefore, although we have not a single ideal medication, the optimal therapy combines ARNI, BB, MRA and SGLT2i to maximize benefits across all disease progression aspects.
